# In silico analysis of non-synonymous single nucleotide polymorphisms (nsSNPs) in the human *GJA3* gene associated with congenital cataract

**DOI:** 10.1186/s12860-020-00252-7

**Published:** 2020-03-06

**Authors:** Mingzhou Zhang, Chen Huang, Zhenyu Wang, Huibin Lv, Xuemin Li

**Affiliations:** 1 Department of Ophthalmology, Peking University Third Hospital, Beijing, China; 2 Beijing Key Laboratory of Restoration of Damaged Ocular Nerve, Peking University Third Hospital, Beijing, China; 3 Medical Research Center, Peking University Third Hospital, Beijing, China

**Keywords:** Congenital cataract, Gap junction protein alpha 3, *GJA3*, Pathogenicity prediction, Bioinformatics

## Abstract

**Background:**

Gap junction protein alpha 3 (*GJA3*), an important pathogenic gene of congenital cataracts, encodes the transmembrane protein connexin46, which functions as an intercellular channel for voltage and chemical gating by forming dodecamers. This study systematically collected nsSNP information for the *GJA3* gene from SNP databases and literature and screened for nsSNPs with high risks of pathogenicity.

**Results:**

A total of 379 nsSNPs of *GJA3* were identified. A total of 88 high-risk pathogenic *GJA3* nsSNPs were found, including 31 published nsSNPs associated with congenital cataracts and 57 novel nsSNPs predicted by all eight online tools. The 88 high-risk pathogenic mutations, which are related to 67 amino acids in the wild-type sequences, cause a decrease in protein stability according to I-Mutant 3.0, MUpro and INPS. G2 and R33 were predicted to participate in post-translational modification and ligand binding by ModPred, RaptorX Binding and COACH. Additionally, high-risk mutations were likely to involve highly conserved sites, random coils, alpha helixes, and extracellular loops and were accompanied by changes in amino acid size, charge, hydrophobicity and spatial structure.

**Conclusions:**

Eighty-eight high-risk pathogenic nsSNPs of *GJA3* were screened out in the study, 57 of which were newly reported. The combination of multiple in silico tools is highly efficient for targeting pathogenic sites.

## Background

The lens is a transparent organ whose main function is to transmit light and focus it on the retina. Gap junctions, formed by docking between lens cells, are responsible for intercellular communication. The lens expresses three gap junction proteins: connexin43 (Cx43, encoded by the *GJA1* gene) is expressed primarily in lens epithelial cells, whereas connexin46 (Cx46, encoded by the *GJA3* gene) and connexin50 (Cx50, ending by the *GJA8* gene) are extensively expressed in lens fibre cells. Cx46 and Cx50 co-localize at gap junction plaques and form mixed hexamers [1, 2]. Accumulating evidence demonstrates that congenital dysfunction of the *GJA3* gene is an important genetic risk factor in autosomal dominant congenital cataracts (ADCCs) [3–5], strongly supporting their close relationship with maintenance of lens transparency [6].

The human *GJA3* gene, mapped on 13q12.11, includes two exons, and exon 2 encodes the 435-amino acid protein Cx46. Cx46 protein contains four transmembrane domains (TM1-TM4), two extracellular loops (E1 and E2), an intracellular loop (CL), and cytoplasmic NH2- and COOH-termini [7]. The two extracellular loops are the most conserved regions and play a crucial role in regulating hemichannel docking [8]. Similar to other connexins, Cx46 functions as an intercellular channel for voltage and chemical gating [9]. After the *Gja3* gene is knocked out, mice present with high calcium influx and dramatically decreasing glutathione in the nucleus, leading to crystalline cleavage and insoluble complex aggregation, eventually, developing into cataracts [10–12]. The first two mutations (N63S and 1137insC) of the *GJA3* gene that cause ADCC were reported by Mackay et al. in 1999 [13]. In 2016, we used targeted exome sequencing to also observe the novel c. 584C > T (p.S195F) missense mutation in the *GJA3* gene causing ADCCs [14].

In the human genome, SNPs are considered responsible for over 90% of sequence variations [15], and play a crucial role in identifying common genetic variants and potential biomarkers for investigating deleterious and neutral effects on protein function associated with numerous diseases or disorders. In protein coding regions, nsSNPs, which might be missense variants, could cause changes in the protein by substitution of amino acids [16]. Over the past few years, using in silico tools to predict deleterious nsSNPs has been an efficient approach requiring less time and cost than experimental procedures, and preliminary screened deleterious nsSNPs are candidates for subsequent functional verification experiments.

The present study aims to combine use of several in silico tools that based on different principles to investigate the potentially detrimental effects of nsSNPs of the *GJA3* gene. Instead of biological experiment confirmation, the study tries to provide a useful method for fast and cost-effective screening for pathologic nsSNPs.

## Results

### nsSNP retrieval

**Fig. 1:**
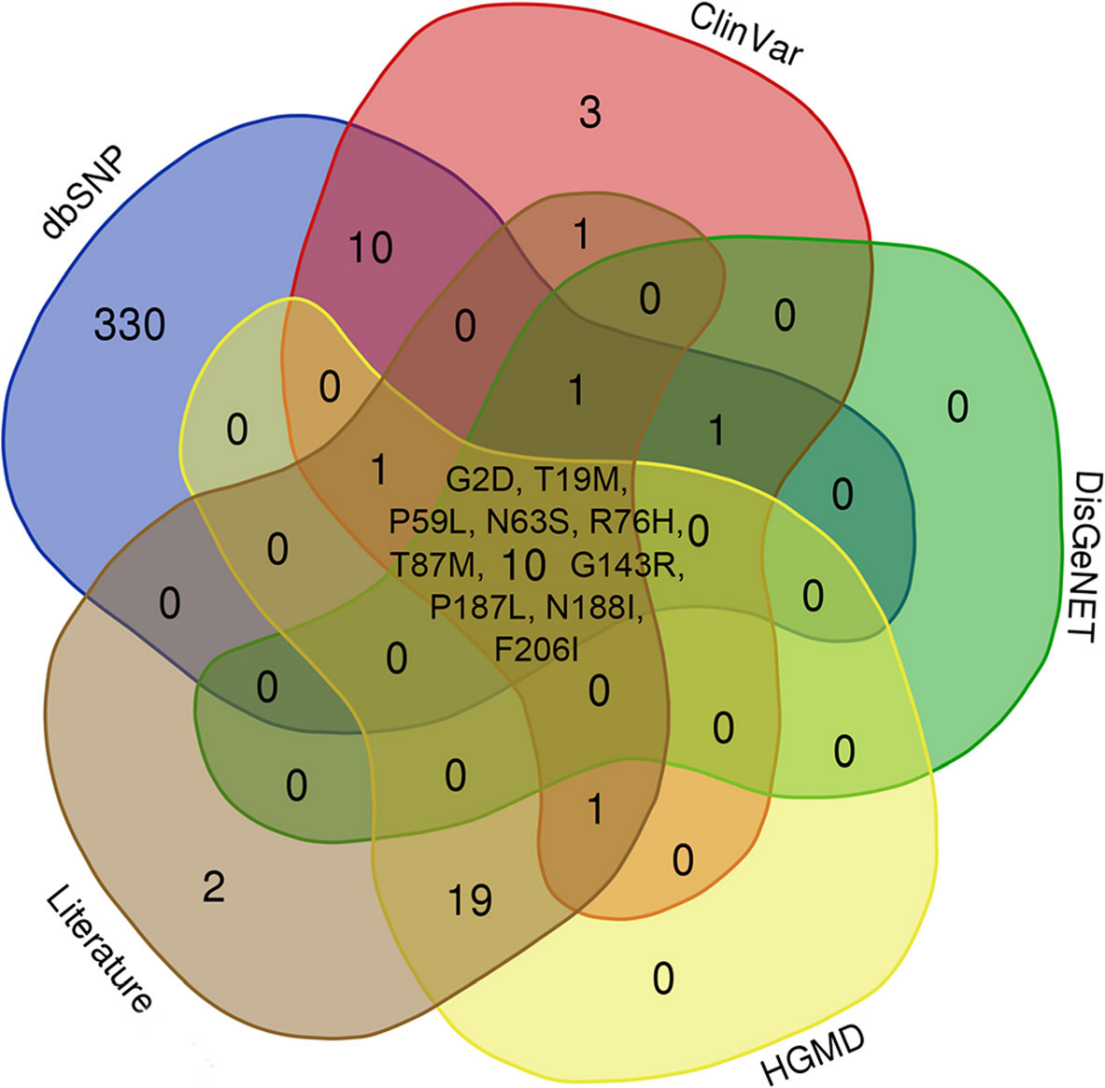
Venn diagram representing the nsSNPs of the *GJA3* gene overlapping in the dbSNP database, ClinVar database, HGMD and DisGeNET database

Four databases were searched by the keyword “GJA3”, and the dbSNP database contained the most nsSNPs (353), followed by the HGMD (31), the ClinVar database (28), and the DisGeNET database (12). Thirty-one nsSNPs were described in the literature as being associated with congenital cataracts, of which only 2 nsSNPs (S195F, E48G) were not found in the above four databases. As shown in Fig. 1, after redundancy was removed, 379 nsSNPs were retrieved for further analyses with only 10 overlapping nsSNPs (G2D, T19M, P59L, N63S, R76H, T87M, G143R, P187L, N188I, F206I) from all four databases and literature.

There 291 nsSNPs contained the information of minor allele frequency (MAF). Except for R133, L299 and G412, other MAFs of nsSNPs were lower than 1% (Additional file 1).

### Predicting deleterious nsSNPs of the *GJA3* gene

**Fig. 2:**
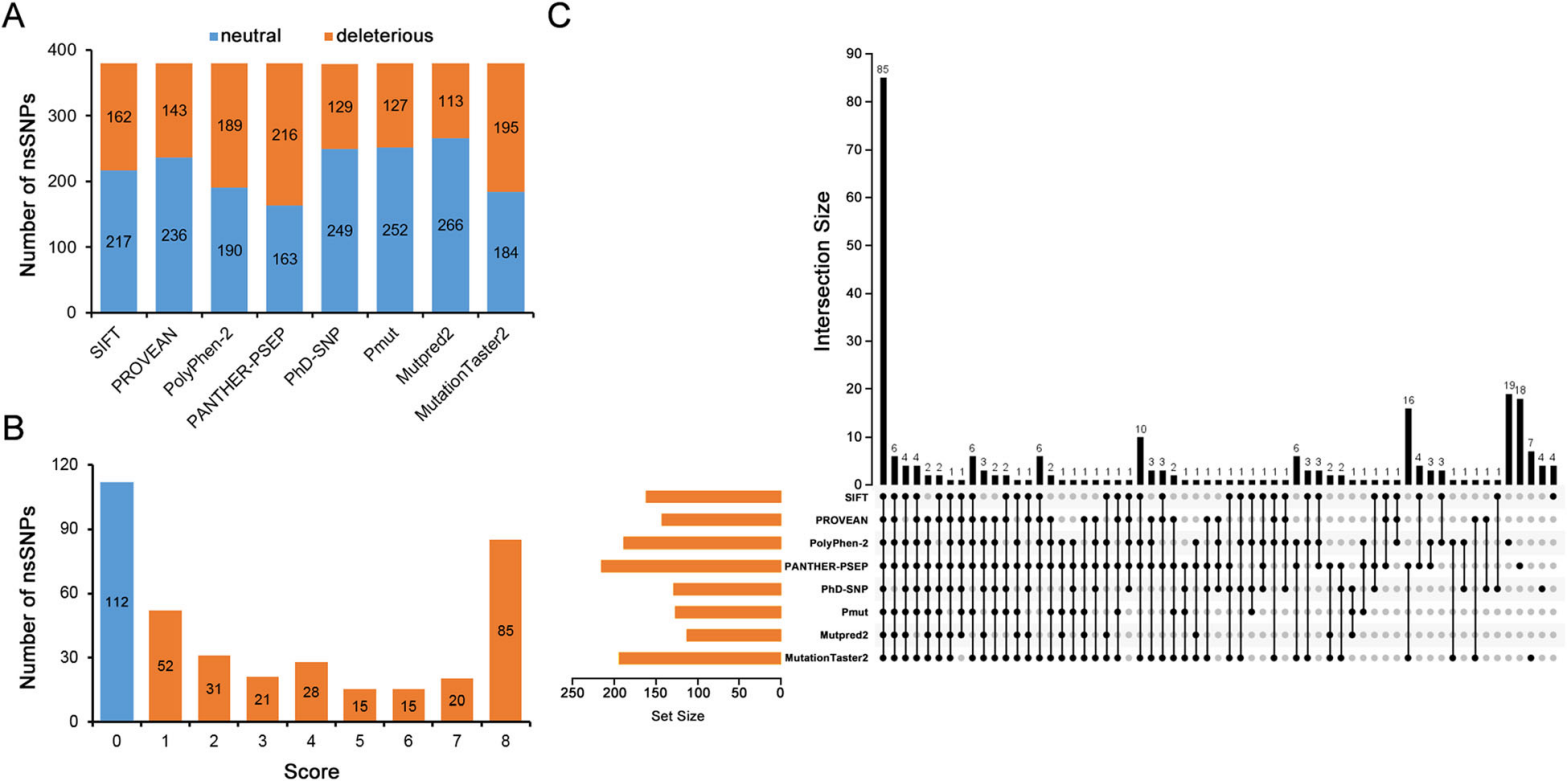
**a** Distribution of deleterious (orange) and neutral (blue) nsSNPs predicted by eight web tools. **b** Deleterious scores of nsSNPs predicted by eight web tools. **c** Matching of deleterious nsSNPs using eight web tools

Multiple approaches were employed to screen the deleterious *GJA3* nsSNPs and identify their structural and functional impacts. A graphical representation of nsSNP prediction by eight web tools is shown in Fig. 2.

Although different options were used, the SIFT, PROVEAN, PhD-SNP, Pmut, MutPred2, and MutationTaster2 tools commonly resulted in binary classification, and these results were then classified into two categories, “neutral” and “deleterious”, in this study (Fig. 2a). Out of 379 nsSNPs, 162 nsSNPs were predicted as “damaging” by SIFT, 143 nsSNPs were predicted as “deleterious” by PROVEAN, 129 nsSNPs were predicted as “disease” by PhD-SNP, 127 nsSNPs were predicted as “disease” by Pmut, 113 nsSNPs were scored higher than 0.5 (suggesting pathogenicity) by MutPred2, and 195 nsSNPs were predicted as “disease causing” by MutationTaster2.

Prediction outcomes of PolyPhen-2 and PANTHER-PSEP were a ternary classification: probably damaging, possibly damaging, or benign (probably benign). PolyPhen-2 predicted 131 nsSNPs (34.6%) as “probably damaging” and 58 nsSNPs (15.3%) as “possibly damaging”, all of which were considered “deleterious”. PANTHER-PSEP predicted 216 nsSNPs to be “deleterious”. Among them, 160 nsSNPs were predicted as “probably damaging”, and the remaining 56 nsSNPs were predicted as “possibly damaging”.

After the results of the above eight computational in silico tools were integrated, intersections between various methods suggested unanimous prediction outputs (Additional file 2). As shown in Fig. 2b and c, 85 nsSNPs were simultaneously predicted as “deleterious” with a score of 8 and thus defined as high-risk nsSNPs, while 112 0-score nsSNPs were suggested as “neutral” nsSNPs. As expected, among 85 high-risk nsSNPs, 28 were reported as causes of congenital cataracts. The remaining three reported nsSNPs obtained high pathogenicity scores. The mutation L11S (score 7) was predicted as a “polymorphism” by MutationTaster2, E62K (score 7) was predicted as “neutral” by PhD-SNP, and N55D (score 5) was predicted as “neutral”, “tolerated”, and “neutral” by SIFT, PROVEAN, and PhD-SNP, respectively.

Although the sensitivity and accuracy of these bioinformatics tools were not perfect, eighty-eight high-risk, deleterious nsSNPs (85 8-score nsSNPs and the 3 remaining reported nsSNPs) screened in this study might provide clues to identify deleterious nsSNPs of *GJA3* and were taken into consideration for further analysis.

### Predicting effects of high-risk nsSNPs on protein stability

**Fig. 3:**
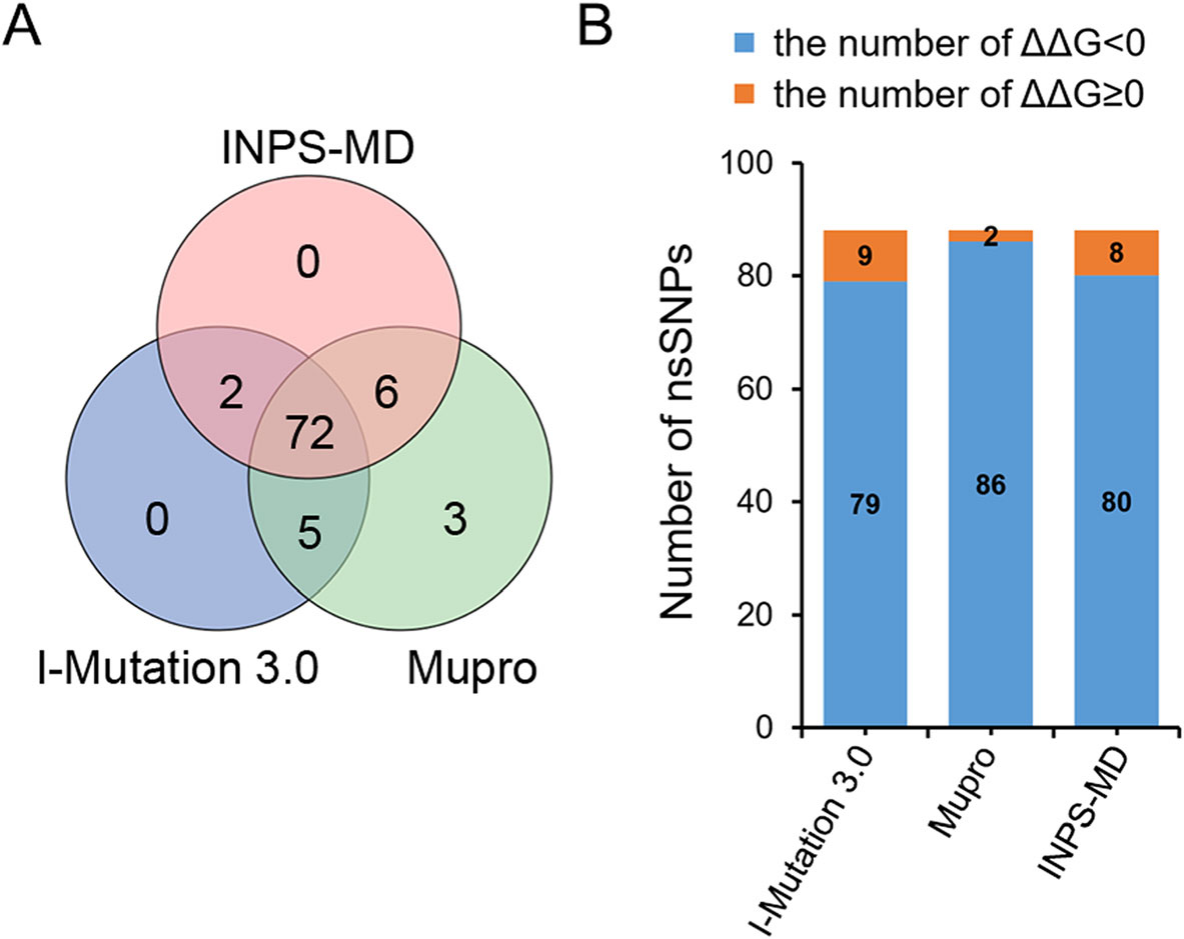
**a** Venn diagram representing the effects of high-risk nsSNPs of *GJA3* on protein stability shared between I-Mutation 3.0, MUpro, and INPS-MD. **b** Distribution of protein stability (ΔΔG < 0, blue and ΔΔG ≥ 0, orange)

The effects of 88 high-risk nsSNPs of *GJA3* on protein stability were predicted using I-Mutant 3.0, MUpro and INPS tools through comparing free energies (Additional file 3 and Fig. 3). A ΔΔG prediction by I-Mutant 3.0 showed that the 79 nsSNPs decreased stability (ΔΔG < 0), whereas 9 nsSNPs increased stability (ΔΔG > 0). Analysed by MUpro and INPS-MD, 86 and 80 nsSNPs were found to decrease protein stability, respectively (Fig. 3a). In total, 72 nsSNPs, including 29 reported pathogenic variants, were predicted as destabilizing; however, no nsSNPs were found to increase protein stability using the three tools (Fig. 3b). The 19 variants L11S, I31F, F32L, R33H, W45S, E48G, R76H, R76G, F77V, I82N, P88S, L146R, F155V, F173L, R183G, V190G, F193S, P197S, and L220Q unanimously showed ΔΔG values less than − 1 kcal/mol calculated by three tools, which would be predicted to disturb the structure and function of the protein.

### Predicting effects of high-risk nsSNPs on post-translational modification sites

To analyse the effect of high-risk nsSNPs in *GJA3* on the PTM of the corresponding protein, the ModPred web server was applied. In the Cx46 protein, 210 amino acid residues were verified to be sites for 16 different modifications with a score of confidence > 0.5 (Additional file 4). As shown in Table 1, 21 residues with 5 modifications were involved in 31 high-risk nsSNPs, including 17 residues (G2, D3, S5, H17, R33, E42, D47, S50, D67, R76, G94, H98, R101, G143, R147, T148, G172) predicted as proteolytic cleavage sites, G2 predicted as an N-terminal acetylation site, T19 predicted as an amidation site, K156 predicted as a SUMOylation site, and P187 and P197 predicted as hydroxylation sites.

**Table 1:** Effect of high-risk nsSNPs in *GJA3* gene on post translational modification sites predicted by ModPred tool

Residue	Modification	Score	Confidence	High-risk nsSNP
G2	N-terminal acetylation	0.53	Low	G2D, G2S
	Proteolytic cleavage	0.52	Low	
D3	Proteolytic cleavage	0.7	Medium	D3H, D3Y
S5	Proteolytic cleavage	0.59	Low	S5R
H17	Proteolytic cleavage	0.51	Low	H17R
T19	Amidation	0.97	High	T19M
R33	Proteolytic cleavage	0.67	Low	R33H, R33P, R33L
E42	Proteolytic cleavage	0.79	Medium	E42A
D47	Proteolytic cleavage	0.62	Low	D47N, D47Y
S50	Proteolytic cleavage	0.75	Medium	S50P
D67	Proteolytic cleavage	0.8	Medium	D67N
R76	Proteolytic cleavage	0.55	Low	R76H, R76G
G94	Proteolytic cleavage	0.67	Low	G94A
H98	Proteolytic cleavage	0.92	High	H98Q
R101	Proteolytic cleavage	0.95	High	R101P
G143	Proteolytic cleavage	0.78	Medium	G143R, G143E
R147	Proteolytic cleavage	0.75	Medium	R147Q, R147W
T148	Proteolytic cleavage	0.55	Low	T148I
K156	SUMOylation	0.52	Low	K156Q
G172	Proteolytic cleavage	0.52	Low	G172D, G172S
P187	Hydroxylation	0.57	Low	P187L, P187S
P197	Hydroxylation	0.64	Medium	P197S

### Predicting effects of high-risk nsSNPs on ligand binding sites

RaptorX Binding and COACH ligand binding site prediction servers were used to predict ligand binding sites in the Cx46 protein. According to the RaptorX Binding server, a pocket multiplicity value greater than 40 indicates an accurate prediction. However, for the Cx46 protein, the largest pocket multiplicity was 39 with a predicted iron (+ 3) cation ligand, which binds to the residues W25, L29, R33, Q81, E160, F163, A211, and S214. The COACH server results show that a cobalt (2+) cation binding the Cx46 protein occupies the rank 1 position with a C-score of 0.15 with aspartate residues substituted at L29, R33, E160, A211, and S214. The rank 2 site binds a zinc (2+) cation at C54, C61, N63, and C65 with a C-score of 0.08. Therefore, the high-risk nsSNPs R33H, R33P, R33L, N63S, Q81P, and A211V were predicted to be significant mutations, as they might affect protein-ligand interactions.

### Phylogenetic conservational analysis of high-risk nsSNPs

Phylogenetic conservation analysis suggested that compared to those in non-conserved regions, amino acids situated in conserved regions were highly damaging. ConSurf predicts amino acids to play structural or functional roles based on conservation and solvent accessibility. Residues are predicted as functional when they are highly conserved and exposed and as structural when they are highly conserved and buried.

**Fig. 4:**
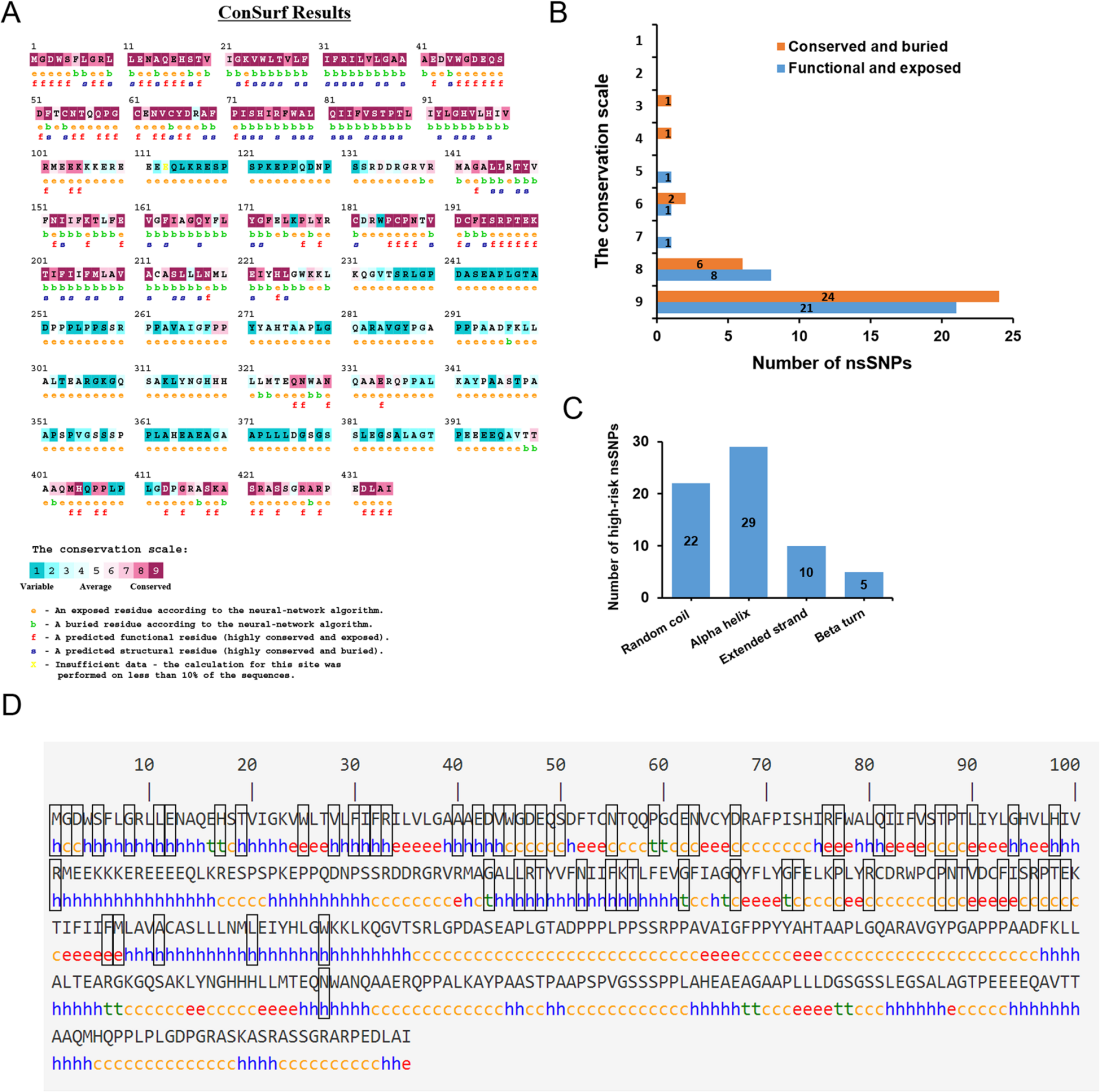
**a** Analysis of evolutionarily conserved amino acid residues of *GJA3* by ConSurf. The colour coding bar shows the conservation score, and boxes indicate the high-risk nsSNPs. **b** Distribution of functional, exposed sites (blue) and conserved, buried sites (orange) of high-risk nsSNPs of *GJA3* predicted by ConSurf. **c** Distribution of high-risk nsSNPs of *GJA3* in random coils, alpha helixes, extended strands and beta turns predicted by SOPMA. **d** SOPMA analysis of the secondary structure of individual amino acid residues in protein produced from the *GJA3* gene. The boxes indicate the high-risk nsSNPs

As shown in Fig. 4a, amino acids 1–105, 141–225, and 401–435 were most conserved, and the remaining locations were more variable. ConSurf results indicate that 88 high-risk nsSNPs refer to 67 amino acids, most of which are highly conserved, including 45 with conservation scores of 9, 14 with scores of 8, and the remaining 8 with scores of 3 to 7. Of the above mentioned 67 amino acid sites, half were predicted as functional residues, while the rest were predicted as structural residues (Fig. 4b, Additional file 5).

### Prediction of amino acid secondary structure of the protein corresponding to *GJA3*

The secondary structure of Cx46 was predicted by SOPMA, which explained the distributions of alpha helix, beta sheet, and coil. The result indicated a large number of random coils (194, 44.60%), followed by 162 alpha helixes (37.24%), 65 extended strands (14.92%) and 14 beta turns (3.22%) in the predicted secondary structure (Fig. 4c). For the 67 amino acid residues that correspond to 88 high-risk nsSNPs, 23 were located in random coils, 29 in alpha helixes, 10 in extended strands, and 5 in beta turns (Fig. 4d).

### Transmembrane protein display of *GJA3*

**Fig. 5:**
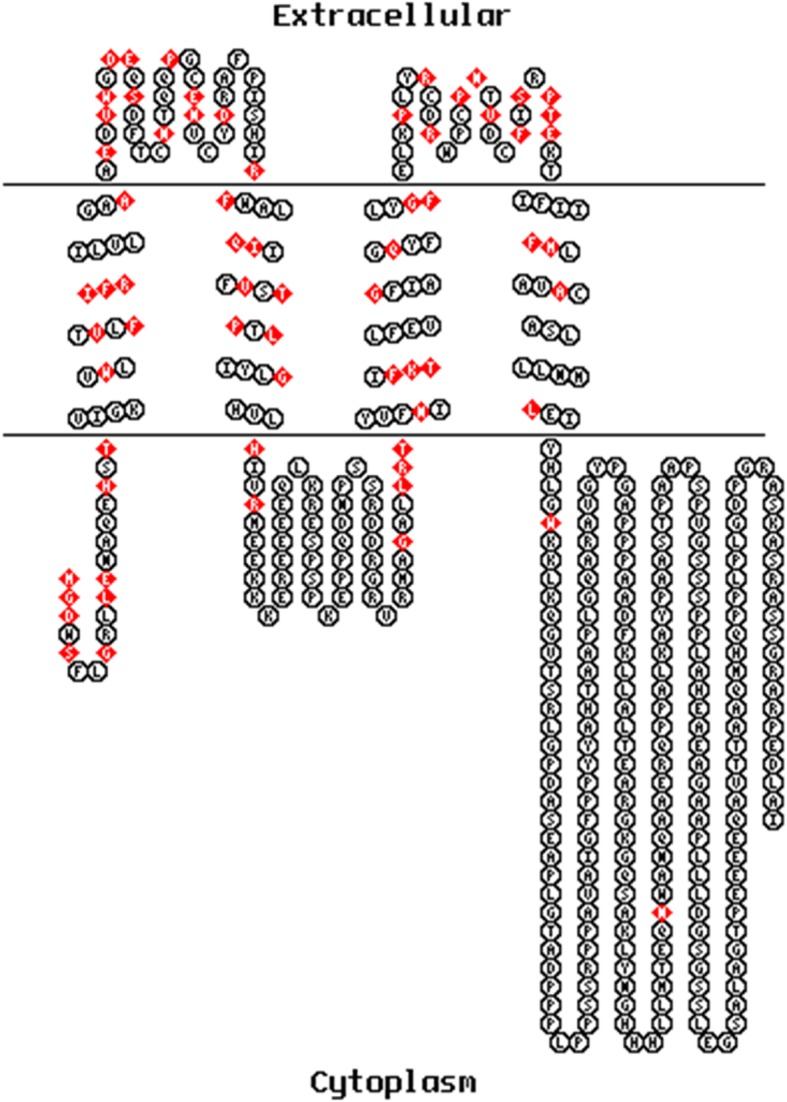
The membrane topological structure of Cx46 was generated based on TMpred using TOPO2. The red dots refer to the high-risk nsSNPs of *GJA3*

TOPO2 was used to display the transmembrane protein expressed by *GJA3* and the locations of the 67 amino acids containing high-risk nsSNPs. Nine nsSNPs occur in the COOH-terminus, 7 in the 1st transmembrane helix, 12 in the 1st extracellular loop, 8 in the 2nd and 3rd transmembrane helixes, 6 in the intracellular loop, 11 in the 2nd extracellular loop, 4 in the 4th transmembrane helix and only 2 in the NH2-terminus (Fig. 5).

### Predicted effects of high-risk nsSNPs on protein properties

HOPE was used to predict the effects of the mutations from 88 high-risk pathogenic nsSNPs of *GJA3* on amino acid size, charge, hydrophobicity, spatial structure and function. Thirty-eight mutated amino acids were smaller than their wild-type counterparts, while 30 mutated amino acids were larger. There were 33 sites with charge changes: 4 changed from neutral to negative, 9 from neutral to positive, 8 from negative to neutral, 11 from positive to neutral, and only 1 from negative to positive. Eighteen mutations reduced hydrophobicity, and 28 increased hydrophobicity. This result suggests that changes in physicochemical properties because of amino acid mutations at these sites lead to changes in protein structure and changes in interactions between protein domains and other molecules, thereby affecting protein function (Additional file 6).

## Discussion

Congenital cataracts are often involved in breakdown of the lens micro-architecture, and most of these cataracts result from gene mutations. Of the cataract protein families for whom the mutant gene is known, approximately 45% show mutations in lens crystallins, 16% in connexins, 12% in various growth or transcription factors, 5% in intermediate filament proteins, 5% in membrane proteins, 5% in the protein degradation apparatus, and approximately 8% in a variety of other functionally divergent genes, including those for lipid metabolism [17]. Cx46 is a member of the connexin family, mainly distributed in lens fibrin, myocardium and kidney, and plays an important role in maintaining lens transparency.

In the present study, 379 nsSNPs in the *GJA3* gene coding region were found in the dbSNP database, ClinVar database, HGMD, and DisGeNET database and related published literature. However, to date, only 10 nsSNPs (G2D, T19M, P59L, N63S, R76H, T87M, G143R, P187L, N188I and F206I) have overlapped among different databases, and only 33 nsSNPs have been published as congenital cataract-causative mutations. Most of known MAFs of nsSNPs in the *GJA3* gene were less than 1%, except for R133, L299 and G412.

SIFT, PROVEAN, PolyPhen-2, PANTHER-PSEP, PhD-SNP, Pmut, MutPred2, and Mutation Taster2 were used to predict the pathogenicity of 379 nsSNPs of *GJA3*, and 88 of them were identified as “*GJA3* gene high-risk pathogenic nsSNPs” with simultaneous harmful predictions by 8 tools and published pathogenic nsSNPs. Twenty-eight of them have been published to be associated with congenital cataracts, and another 57 are novel high-risk nsSNPs. The other 3 published nsSNPs (L11S, N55D, E62K) were scored 7, 5 and 7, respectively, and thus were considered disease-causing by four of eight software methods (PolyPhen-2, MutPred2, PANTHER-PSEP and Pmut). In the deleterious prediction, nsSNPs with high MAFs obtained lower scores (R133P, R133Q 2, L299M and G412R got 4, 2, 0 and 1 points, respectively), which indicated was consistent with the past understanding [18, 19].

Different computational methods present various prediction characteristics based on different databases. A previous study, which compared 12 objective indicators from 23 tools based on three independent benchmark datasets, shows that Mutation Taster, Polyphen2 and SIFT present high sensitivity based on ClinVar benchmark data. In the meanwhile, compared to other tools, Mutation Taster, Polyphen2 and SIFT present higher sensitivity, while PROVEAN shows consistently high specificity based on ClinVar, TP53 and PPARG benchmark data, ranging from 65.00 to 76.99% [20]. Additionally, SIFT, PolyPhen2 and PROVEAN present high values in AUC, high-specificity AUC and high-sensitivity AUC (area under the curve) [20]. Consequently, Mutation Taster 2, Polyphen2, SIFT and PROVEAN were brought into prediction. In order to increase the polymorphism of calculation methods and databases involved, PANTHER-PSEP, PhD-SNP, PMut and MutPred2 were also included.

The stability of proteins is critical to their biological function, activity and regulation of biomolecules. Incorrect folding and decreased stability are the major consequences of pathogenic missense mutations [21, 22]. The folding free energy (ΔG) is used to measure the thermodynamic stability of proteins and equals the difference in free energy between folded and unfolded states. Both the wild type and mutants type have their own ΔG values, and the difference between them is the folding free energy change (ΔΔG), which is calculated by the equation ΔΔG value = ΔG (mutant protein) – ΔG (wild-type protein) in kcal/mol at pH 7 and 25 °C. In general, ΔΔG > 0 is equivalent to increased stability in the mutant protein, while ΔΔG < 0 indicates a decrease in stability. Out of 88 high-risk nsSNPs, 72 were calculated to decrease protein stability by I-mutation 3.0, MUpro and INPS, and the remaining 16 nsSNPs showed negative ΔΔG values according to at least one of the methods I-mutation 3.0, MUpro and INPS. However, one should be cautious when analysing the mutations based on ΔΔG. Whether a mutation with a ΔΔG other than zero causes significant structural changes in the protein depends on the relative values of ΔG and ΔΔG [23]. A mutation that leads to a small magnitude of ΔΔG may not result in a significant structural change in a protein with a large ΔG. In addition, some harmful mutations can be stabilizing, which indicates that predicting pathogenicity through a single method is very uncertain [24].

Conformational changes are required for the function of many proteins [25]; therefore, conformational flexibility and rigidity must be finely balanced [26]. The high-risk nsSNPs R33H, R33P, R33L, N63S, Q81P and A211V were predicted as ligand binding sites by RaptorX Binding and COACH ligand binding site prediction servers, all of which were confirmed as highly conserved with a 9 score by ConSurf, which could be used in screening deleterious mutations because neutral nsSNPs are more common in variable positions, while the deleterious nsSNPs are more frequent in conserved positions [27].

According to SOPMA secondary structure calculations, 88 high-risk nsSNPs were located in 66 amino acid sites, and 77.27% of sites were located in alpha helixes and random coils, which is in accordance with the previous recognition that both harmful and polymorphic mutations are mainly located in helixes and coil regions and not frequently in β turns [23]. Proteins populate a range of conformations instead of being static. Regional flexibility mainly depends on the local residue microenvironment and side chain lengths [23]. As shown in Additional file 6, the wild-type amino acid glycine is flexible enough to twist, and the mutations in G2, G94, G143, G162, and G172 are all highly harmful. In addition, past analysis indicated that compared with mutations on the surface, mutations fully or partially buried tend to be more harmful. Consistent with this observation, the wild-type residues E12, R33, A40, V44, E48, P59, D67, V85, P88, L90, S197, and A211 are all buried in the core of the protein, while the corresponding mutated residues were not fit for the size changes. In addition, R33 and A211 were predicted as binding sites, which implies that conformational changes occur when proteins interact.

**Fig. 6:**
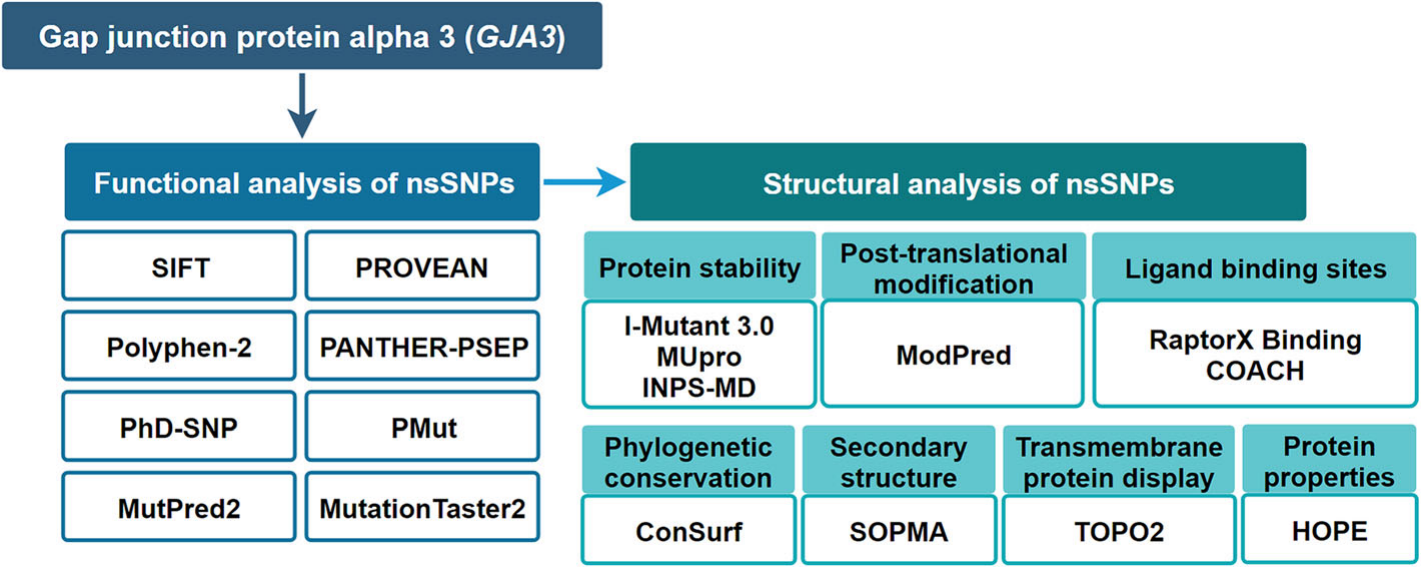
Diagrammatic representation of the *GJA3* gene in silico work flow used to analyse the deleterious nsSNPs and perform their structural modelling analysis

In most cases, proteins perform biological functions as temporary or permanent complexes by interacting with other macromolecules. Cx46 functions as a transmembrane hexamer that interacts with a similar structure docking on the neighbouring cells. In addition, Cx46 can form into a heteromeric and heterotypic intercellular dodecamer with connexin50 in the lens [28]. The dodecamer plays an important role in maintaining eye lens transparency as an intercellular channel to deliver various chemical messages and remove metabolic waste by passing ions, metabolites, hormones, and other small signalling molecules [29]. Therefore, mutations in or near some special amino acids that contribute to the functional spatial conformation are at a high risk of causing pathologies. Missense mutations result in the substitution of amino acids and consequent changes in amino acid size, charge and hydrophobicity, which may disturb protein folding and interaction. According to the analysis from HOPE, the changes associated with mutations would lead to either loss of interactions or structural perturbations, especially in the transmembrane domains. Additionally, the introduction or loss of charge or hydrophobicity would cause repulsion, misfolding or loss of interactions. D3 is critical for polarization and trans-junctional voltage, and the substitution of D3 leads to obstruction of gating [30, 31]. W4, L7, I10, L11 and V14 in the NH2-terminus participate in the formation of the hydrophobic face with the NH2-terminus [32]. Consistent with this finding, several mutations near those sites were predicted to result in a high risk of pathology, as shown in Fig. 6.

Although it is more reliable to distinguish pathogenic mutations through experiments, it takes much time to perform repeated experiments on all nsSNPs. Different methods present a certain degree of consistency for hazard prediction. The methods in the current study offer clues to the various effects of mutations, which were used to describe pathogenicity. However, there are some limitations in the study. First, the reported causative nsSNPs were limited in number, and the prediction results have not been verified by laboratory, so the PPV, NPV, sensitivity, specificity and accuracy for the *GJA3* gene were hardly calculated. Thus, the results of the prediction could only be considered as a reference resource. The second limitation is that the pathogenic analysis is based on public data. It is difficult to acquire additional clinical or heredity information behind each nsSNP except for those published. The third is that there are some overlaps of the disease prediction mechanism among different in silico tools because most of them were based on changes in conserved residues over time.

## Conclusions

In this study, out of 88 predicted high-risk pathogenic nsSNPs, 57 were novel sites involved in the pathogenesis of congenital cataracts. Combinations of multiple in silico tools provide many more dimensions to predict the effects of mutations on proteins, which could be a cost-effective and fast screening method to further guide diagnostic and experimental strategies. Nevertheless, in silico tools cannot replace conclusive experiments, and their results should be verified by further biology verification.

## Methods

The deleterious nature of variations in the structure, stability and function of the Cx46 protein was predicted using various in silico tools. An overview of the computational methods used in the present study is depicted in Fig. 6.

### Data retrieval of nsSNPs

The nsSNP distribution of the *GJA3* gene was collected from the dbSNP database (http://www.ncbi.nlm.nih.gov/projects/SNP/) [33], the ClinVar database (https://www.ncbi.nlm.nih.gov/clinvar) [34] of the National Center for Biotechnology Information (NCBI), the Human Gene Mutation Database (HGMD, http://www.hgmd.cf.ac.uk/ac/index.php) [35], and the DisGeNET database (http://www.disgenet.org) [36] using limits of “*Homo sapiens*” and “coding nonsynonymous”. The previous literature was also reviewed. The amino acid and DNA sequences, SNP IDs, wild-type amino acids, amino acid positions, missense amino acids, minor allele frequency (MAF), and other information were collected.

### Prediction of deleterious nsSNPs

In the present study, eight web tools were used to predict the functional impact and pathogenic nature of nsSNPs. All tools were used according to their default settings if not stated otherwise.

#### SIFT

SIFT [37] (Sorting Intolerant From Tolerant, https://sift.bii.a-star.edu.sg/) predicts whether an amino acid substitution causes deleterious based on sequence homology and the physical properties of amino acids. A missense variant is predicted to be deleterious, when the SIFT score < 0.05, while a score ≥ 0.05 indicated that a variant is benign.

#### PROVEAN

PROVEAN [38] (Protein Variation Effect Analyzer, http://provean.jcvi.org/index.php) is a tool for predicting the functional effect of amino acid substitutions, insertions and deletions, that introduces a delta alignment score of a protein query sequence to measure the effect of a variation. High delta scores are considered as variations with neutral effects, while low delta scores are considered as amino acid variations with negative effects on protein function. In order to provide binary predictions, the cutoff value of PROVEAN scores is set to 2.5 to obtain high balanced accuracy.

#### PolyPhen2

PolyPhen2 [39] (Polymorphism Phenotyping v2, http://genetics.bwh.harvard.edu/pph2/), which uses the HumVar and HumDiv datasets and is based on a naïve Bayes classifier trained by supervised machine learning. An iterative greedy algorithm was used to selected predictive features, including eight sequence-based and three structure-based features, through which different mutations are categorized as “probably damaging”, “possibly damaging”, or “benign”.

#### PANTHER-PSEP

PANTHER-PSEP [40] (PANTHER -position-specific evolutionary preservation, http://pantherdb.org/tools/csnpScoreForm.jsp) uses a metric relevant but different from ‘evolutionary preservation’: the possible sequences of ancestral proteins at nodes in a phylogenetic tree are reconstructed based on homologous proteins. From current state of each amino acid, its history can be traced back to calculate the duration that amino acid has been preserved in its ancestors. The PSEP score was classified as “probably damaging” (the preservation time > 450 my), “possibly damaging” (200 my < the preservation time < 450 my) and “probably benign” (the preservation time < 200 my).

#### PhD-SNP

PhD-SNP [41] (Predictor of human Deleterious Single Nucleotide Polymorphisms, http://snps.biofold.org/phd-snp/phd-snp.html), which is simply designed, is supported by a machine-learning core and based on comparative conservation scores of multiple sequence alignments. PhD-SNP was trained and tested based on the ClinVar dataset, which contains about ∼36,000 deleterious and benign SNVs, identifies a SNP effect as a disease or neutral and gives a reliability index score.

#### PMut

PMut [42] (http://mmb.irbbarcelona.org/PMut/) was trained and tested by the manually created database SwissVar (October 2016 release), which includes 27,203 harmful and 38,078 benign mutations for 12,141 proteins. The prediction scores of PMut are from 0 to 1, and the cutoff value is set to 0.5 (neutrual, 0 to 0.5; pathological, 0.5 to 1).

#### MutPred2

MutPred2 [43] (http://mutpred.mutdb.org/) is a machine learning-based software package that analyses the inferences of structural, functional and phenotypic consequences of sequence variants. It was trained and tested on 53,180 deleterious and 206,946 unlabelled (assumed benign) variants collected from the HGMD, the SwissVar database, the dbSNP database and inter-species pairwise alignments. A missense mutation with a MutPred2 score > 0.5 is considered “harmful”.

#### MutationTaster2

MutationTaster2 [44] (http://www.mutationtaster.org/), which combines numerical publicly available SNPs from Genomes Projects, ClinVar and HGMD, was designed to predict the functional effects of amino acid mutations and variations across intron-exon borders. Variants were categorized as a “polymorphism” or “disease causing”.

To integrate the predictive results of eight web tools, the results were classified into two categories: “neutral” and “deleterious”. Results of “benign”, “tolerated”, “polymorphism”, “probably benign”, and “harmless” were categorized into “neutral” with a score of 0; meanwhile, the results “pathogenic”, “deleterious”, “possibly damaging”, “probably damaging”, “disease causing” or “harmful” were categorized into “deleterious” with a score of 1. Intersections between various methods were analysed using TBtools. The 8-score nsSNPs and the nsSNPs reported in previous studies were defined as “high-risk nsSNPs”.

### Predicting effects of nsSNPs on protein stability

I-Mutant 3.0, MUpro and INPS-MD were used to evaluate the protein stability changes of Cx46 caused by the high-risk nsSNPs of the *GJA3* gene.

#### I-mutant 3.0

I-Mutant 3.0 [45] (http://gpcr2.biocomp.unibo.it/cgi/predictors/I-Mutant3.0/I-Mutant3.0.cgi) was trained and tested on a ΔΔG Mut dataset obtained from ProTherm. The predictor can estimate the stability change, which is measured by ΔΔG value (kcal/mol), upon single-site mutation based on a protein structure or a protein sequence. A ΔΔG value less than ‘0’ indicates that the variant decreases the protein stability. On the contrary, a ΔΔG value greater than 0 indicates that the variant elevates the protein stability.

#### MUpro

MUpro [46] (http://mupro.proteomics.ics.uci.edu/), based on support vector machines and neural networks machine learning methods, which can be used to predict the effects of single-site amino acid mutations on protein stability. MUpro can predict protein stability changes merely using sequence information or combining that information with tertiary structure. The cut-off value of ΔΔG is same to I-mutant 3.0.

#### INPS-MD

INPS-MD [47, 48] (Impact of Non-synonymous mutations on Protein Stability-Multi Dimension, https://inpsmd.biocomp.unibo.it) is a method used to predict stability of protein variants from sequences and structures. The INPS-MD predictor using sequences is based on a simplified support vector (SVR) as implemented by the libsvm package, which was only tested by linear and radial basis function (RBF) kernels. INPS-MD predictions can be interpreted to identify stabilizing (ΔΔG > 0) and destabilizing (ΔΔG < 0) variations.

### Prediction of post-translational modification sites

ModPred [49], which based on sequence, is used to predict potential post-translational modification (PTM) sites in proteins. It consists of 34 ensembles of logistic regression models trained separately on a combined set of 126,036 non-redundant experimentally verified sites for 23 different modifications that were obtained from public databases and an ad hoc literature search. The Cx46 protein sequence in FASTA format was used as input to predict various PTM sites.

### Prediction of ligand binding sites

The ligand binding sites in Cx46 were predicted by using the RaptorX Binding server and the COACH server.

#### RaptorX

RaptorX Binding [50] (http://raptorx.uchicago.edu/BindingSite/) is a web portal for predicting the binding sites of a protein sequence based upon a 3D model predicted by RaptorX. RaptorX predicts protein secondary and tertiary structures, contact and distance maps, solvent accessibility, disordered regions, functional annotation and binding sites. For binding site prediction, one measure of pocket multiplicity, in addition to *P*-value, uGDT (GDT), and uSeqID (SeqID), is used to judge the quality of a predicted pocket. The higher the score is, the more accurate the predicted pocket, especially when the score is over 40.

#### COACH

COACH [51, 52] (https://zhanglab.ccmb.med.umich.edu/COACH/) is a meta-server approach to protein-ligand binding site prediction using two comparative methods, TM-SITE and S-SITE, which recognize ligand-binding templates from the BioLiP protein function database by binding-specific substructure and sequence profile comparisons. In the COACH server, the top 10 models were ranked by the cluster size and given a C-score, and their PDB hits, ligand names, available downloadable complex structures, and consensus binding residues were given. The predicted C-scores lie between 0 and 1, where the scores increase with reliability.

### Phylogenetic conservation analysis

The ConSurf web server [53] (http://consurf.tau.ac.il) analyses the evolutionary pattern of the amino/nucleic acids of the macromolecule to reveal areas important for function and/or structure. The corresponding conservation score ranges from 1 to 9, where 1 indicates rapidly evolving (variable) regions, 5 indicates regions that are evolving mildly, and 9 indicates conserved positions. Exposed residues with high scores are thought to be functional residues, whereas buried residues with high scores are considered structural.

### Prediction of the amino acid secondary structure produced from the *GJA3* gene

SOPMA [54] is an advanced version of the self-optimized prediction method (SOPM), which can predict the secondary structure(α helix, β turn and coil) of 69.5% of amino acids in the entire database containing 126 non-homologous (less than 25% homologous) protein chains. The SOPMA and a neural network method (PHD) jointly correctly predicts 82.2% of residues for 74% of co-predicted amino acids.

### Prediction of high-risk nsSNPs effects on protein structure

#### TOPO2

TOPO2 (http://www.sacs.ucsf.edu/TOPO2/), which is a simple graphics program, was used to create images of transmembrane protein according to the sequences.

#### HOPE

HOPE [55] (http://www.cmbi.ru.nl/hope/) can build an automatic mutant analysis server that can provide insight into the structural effects of a mutation. Structural information was collected from a series of sources, including calculations on the 3D protein structure, sequence annotations in UniProt and prediction from Reprof software. The program Yasara, with an automatic modelling script only needing the sequence of the protein of interest, was used to build a homology model when possible.

## Supplementary information

**Additional file 1. **The nsSNPs collections of *GJA3* gene from dbSNP database, the HGMD, the ClinVar database, the DisGeNET database and literatures.

**Additional file 2. **Deleterious predictions of nsSNPs of *GJA3* gene by eight computational in silico tools.

**Additional file 3. **The effects of 88 high-risk nsSNPs of *GJA3* gene on protein stability predicted by I-Mutant 3.0, MUpro and INPS tools.

**Additional file 4.** Predictions of post-translational modification sites in GJA3 protein by ModPred.

**Additional file 5.** Residues predictions of 67 amino acids by ConSurf.

**Additional file 6. **Predicted effects of the mutations from 88 high-risk pathogenic nsSNPs of *GJA3* on amino acid size, charge, hydrophobicity, spatial structure and function.

## Data Availability

All data generated or analysed during this study are included in this published article and its Additional files.

## Abbreviations

ADCC: Autosomal dominant congenital cataract
AUC: Area under the curve
CL: Intracellular loop
Cx: Connexin
E: Extracellular loop
GJA3: Gap junction protein alpha 3
HGMD: Human gene mutation database
hser-AUC: High-sensitivity regional area under the curve
hspr-AUC: High-specificity regional area under the curve
MAF: Minor allele frequency
NCBI: National Center for Biotechnology Information
nsSNP: Non-synonymous single nucleotide polymorphism
PhD-SNP: Predictor of human deleterious single nucleotide polymorphisms
PolyPhen2: Polymorphism phenotyping v2
PROVEAN: Protein variation effect analyzer
PSEP: Position-specific evolutionary preservation
PTM: Potential post-translational modification
RBF: Radial basis function
SOPM: Self-optimized prediction method
SVR: Simplified support vector
TM: Transmembrane domain
