# Supplementary material for: In silico analysis of non-synonymous single nucleotide polymorphisms (nsSNPs) in the human GJA3 gene associated with congenital cataract
Source: BMC Mol Cell Biol. 2020 Mar 6;21:12. doi: 10.1186/s12860-020-00252-7 (PMC7060521; doi:10.1186/s12860-020-00252-7)
Supplement: Supplementary file 6 — Additional file 6. Predicted effects of the mutations from 88 high-risk pathogenic nsSNPs of GJA3 on amino acid size, charge, hydrophobicity, spatial structure and function. [file 12860_2020_252_MOESM6_ESM.docx]

Additional file 6. Predicted effects of the mutations from 88 high-risk pathogenic nsSNPs of *GJA3* on amino acid size, charge, hydrophobicity, spatial structure and function.

| Mutation | Domains | ConSurf  Conservation score | SOPMA Secondary structure | Amino acids size | Charge | Hydrophobicity | Overall impact |
| --- | --- | --- | --- | --- | --- | --- | --- |
| M1I | - | 9 | α helix | W>M | - |  | The mutant residue is smaller, this might lead to loss of interactions. |
| M1V | - | 9 | α helix | W>M | - |  | The mutant residue is smaller, this might lead to loss of interactions. |
| G2D | Intramembrane, NH2-termini | 8 | random coil | W<M | Neu→Neg | ↓ | The torsion angles for this residue are unusual. Only glycine is flexible enough to make these torsion angles, mutation into another residue will force the local backbone into an incorrect conformation and will disturb the local structure. |
| G2S | Intramembrane, NH2-termini | 8 | random coil | W<M | - |  | The mutant residue is bigger, this might lead to bumps. The torsion angles for this residue are unusual. Only glycine is flexible enough to make these torsion angles, mutation into another residue will force the local backbone into an incorrect conformation and will disturb the local structure. |
| D3H | Intramembrane, NH2-termini | 9 | random coil | W<M | Neg→Neu |  | The charge of the wild-type residue is lost by this mutation. This can cause loss of interactions with other molecules. The residue is located on the surface of the protein, mutation of this residue can disturb interactions with other molecules or other parts of the protein. |
| D3Y | Intramembrane, NH2-termini | 9 | random coil | W<M | Neg→Neu | ↑ | The charge of the wild-type residue is lost by this mutation. This can cause loss of interactions with other molecules. The residue is located on the surface of the protein, mutation of this residue can disturb interactions with other molecules or other parts of the protein. |
| S5R | Intramembrane, NH2-termini | 8 | α helix | W<M | Neu→Pos | ↓ | The mutation introduces a charge at this position, this can cause repulsion between the mutant residue and neighboring residues. The residue is located on the surface of the protein, mutation of this residue can disturb interactions with other molecules or other parts of the protein. The mutation might cause loss of hydrophobic interactions with other molecules on the surface of the protein. |
| G8E | Intramembrane, NH2-termini | 8 | α helix | W<M | Neu→Neg | ↓ | The mutation introduces a charge at this position, this can cause repulsion between the mutant residue and neighboring residues.  The residue is located on the surface of the protein, mutation of this residue can disturb interactions with other molecules or other parts of the protein. |
| L11S | Intramembrane, NH2-termini | 9 | α helix | W>M |  | ↓ | The mutation will cause an empty space in the core of the protein. The mutation will cause loss of hydrophobic interactions in the core of the protein. |
| E12K | Intramembrane, NH2-termini | 9 | α helix | W<M | Neg→Pos |  | The charge of the buried wild-type residue is reversed by this mutation, this can cause repulsion between residues in the protein core. The wild-type residue was buried in the core of the protein. The mutant residue is bigger and probably will not fit. |
| H17R | Topological domain, Cytoplasmic, NH2-termini | 9 | β turn | W<M | Neu→Pos |  | The mutation introduces a charge at this position, this can cause repulsion between the mutant residue and neighboring residues. The residue is located on the surface of the protein, mutation of this residue can disturb interactions with other molecules or other parts of the protein. |
| T19M | Topological domain, Cytoplasmic, NH2-termini | 9 | α helix | W<M |  | ↑ | The mutant residue is bigger, this might lead to bumps. The mutation introduces a more hydrophobic residue at this position. This can result in loss of hydrogen bonds and/or disturb correct folding. |
| W25L | 1^st^ transmembrane domain | 9 | extended strand | W>M |  |  | The mutant residue is smaller than the wild-type residue. This will cause a possible loss of external interactions. |
| V28M | 1^st^ transmembrane domain | 8 | α helix | W<M |  |  | The residue is located on the surface of the protein, mutation of this residue can disturb interactions with other molecules or other parts of the protein. |
| F30L | 1^st^ transmembrane domain | 9 | α helix | W>M |  |  | This will cause a possible loss of external interactions. |
| I31F | 1^st^ transmembrane domain | 9 | α helix | W<M |  |  | The residue is located on the surface of the protein, mutation of this residue can disturb interactions with other molecules or other parts of the protein. |
| F32L | 1^st^ transmembrane domain | 9 | α helix | W>M |  |  | This will cause a possible loss of external interactions. |
| R33H | 1^st^ transmembrane domain | 9 | α helix | W>M | Pos→Neu |  | The charge of the buried wild-type residue is lost by this mutation. The mutation will cause an empty space in the core of the protein. |
| R33L | 1^st^ transmembrane domain | 9 | α helix | W>M | Pos→Neu | ↑ | This differences in hydrophobicity can affect the hydrophobic interactions with the membrane lipids. The charge of the buried wild-type residue is lost by this mutation. The mutation will cause loss of hydrogen bonds in the core of the protein and as a result disturb correct folding. |
| R33P | 1^st^ transmembrane domain | 9 | α helix | W>M | Pos→Neu | ↑ | This differences in hydrophobicity can affect the hydrophobic interactions with the membrane lipids. The mutation will cause an empty space in the core of the protein. The mutation will cause loss of hydrogen bonds in the core of the protein and as a result disturb correct folding. |
| A40T | 1^st^ transmembrane domain | Transmembrane 1 | α helix | W<M |  | ↑ | The wild-type residue was buried in the core of the protein. The mutant residue is bigger and probably will not fit. The mutation will cause loss of hydrophobic interactions in the core of the protein. |
| A40 V | 1^st^ transmembrane domain | Transmembrane 1 | α helix | W<M |  |  | The wild-type residue was buried in the core of the protein. The mutant residue is bigger and probably will not fit. |
| E42A | 1^st^ extracellular loop | Transmembrane 1 | α helix | W>M | Neg→Neu | ↑ | The charge of the wild-type residue is lost by this mutation. This can cause loss of interactions with other molecules. |
| V44L | 1^st^ extracellular loop | 9 | α helix | W<M |  |  | The wild-type residue was buried in the core of the protein. The mutant residue is bigger and probably will not fit. |
| V44M | 1^st^ extracellular loop | 9 | α helix | W<M |  |  | The wild-type residue was buried in the core of the protein. The mutant residue is bigger and probably will not fit. |
| W45S | 1^st^ extracellular loop | 9 | random coil | W>M |  | ↓ | The mutation will cause an empty space in the core of the protein. The mutation will cause loss of hydrophobic interactions in the core of the protein. |
| D47N | 1^st^ extracellular loop | 9 | random coil |  | Neg→Neu |  | The charge of the wild-type residue is lost by this mutation. This can cause loss of interactions with other molecules. |
| D47Y | 1^st^ extracellular loop | 9 | random coil | W<M | Neg→Neu | ↑ | The charge of the wild-type residue is lost by this mutation. This can cause loss of interactions with other molecules. The residue is located on the surface of the protein, mutation of this residue can disturb interactions with other molecules or other parts of the protein. |
| E48G | 1^st^ extracellular loop | 9 | random coil | W>M | Neg→Neu | ↑ | The charge of the buried wild-type residue is lost by this mutation. The mutation will cause an empty space in the core of the protein. The mutation will cause loss of hydrogen bonds in the core of the protein and as a result disturb correct folding. |
| S50P | 1^st^ extracellular loop | 9 | random coil | W<M |  | ↑ | The residue is located on the surface of the protein, mutation of this residue can disturb interactions with other molecules or other parts of the protein. |
| N55D | 1^st^ extracellular loop | 9 | extended strand |  | Neu→Neg |  | The mutant residue introduces a charge in a buried residue which can lead to protein folding problems. |
| P59L | 1^st^ extracellular loop | 9 | β turn | W<M |  |  | The wild-type residue was buried in the core of the protein. The mutant residue is bigger and probably will not fit. |
| E62K | 1^st^ extracellular loop | 7 | extended strand | W<M | Neg→Pos |  | The mutation introduces the opposite charge at this position. This possibly disrupts contacts with other molecules. The residue is located on the surface of the protein, mutation of this residue can disturb interactions with other molecules or other parts of the protein. |
| N63S | 1^st^ extracellular loop | 9 | random coil | W>M |  | ↑ | The mutation will cause an empty space in the core of the protein. The mutation will cause loss of hydrogen bonds in the core of the protein and as a result disturb correct folding. |
| D67N | 1^st^ extracellular loop | 9 | random coil |  | Neg→Neu |  | The charge of the buried wild-type residue is lost by this mutation. |
| R76G | 2^nd^ transmembrane domain | 9 | extended strand | W>M | Pos→Neu | ↑ | The charge of the wild-type residue will be lost, this can cause loss of interactions with other molecules or residues. The mutant residue is smaller, this might lead to loss of interactions. The mutation introduces a more hydrophobic residue at this position. This can result in loss of hydrogen bonds and/or disturb correct folding. |
| R76H | 2^nd^ transmembrane domain | 9 | extended strand | W>M | Pos→Neu |  | The charge of the wild-type residue will be lost, this can cause loss of interactions with other molecules or residues. The mutant residue is smaller, this might lead to loss of interactions. |
| F77V | 2^nd^ transmembrane domain | 8 | extended strand | W>M |  |  | The mutant residue is smaller, this might lead to loss of interactions. |
| Q81P | 2^nd^ transmembrane domain | 9 | α helix | W>M |  | ↑ | The mutant residue is smaller, this might lead to loss of interactions. The mutation introduces a more hydrophobic residue at this position. This can result in loss of hydrogen bonds and/or disturb correct folding. |
| I82N | 2^nd^ transmembrane domain | 9 | extended strand | W<M |  | ↓ | The mutant residue is bigger, this might lead to bumps. This differences in hydrophobicity can affect the hydrophobic interactions with the membrane lipids. |
| V85L | 2^nd^ transmembrane domain | 9 | extended strand | W<M |  |  | The wild-type residue was buried in the core of the protein. The mutant residue is bigger and probably will not fit. |
| V85M | 2^nd^ transmembrane domain | 9 | extended strand | W<M |  |  | The wild-type residue was buried in the core of the protein. The mutant residue is bigger and probably will not fit. |
| T87A | 2^nd^ transmembrane domain | 9 | random coil | W>M |  | ↑ | This will cause a possible loss of external interactions. |
| T87M | 2^nd^ transmembrane domain | 9 | random coil | W<M |  | ↑ | The residue is located on the surface of the protein, mutation of this residue can disturb interactions with other molecules or other parts of the protein. |
| P88L | 2^nd^ transmembrane domain | 9 | random coil | W<M |  |  | The wild-type residue was buried in the core of the protein. The mutant residue is bigger and probably will not fit. |
| P88S | 2^nd^ transmembrane domain | 9 | random coil | W>M |  | ↓ | The mutation will cause an empty space in the core of the protein. The hydrophobicity of the wild-type and mutant residue differs. The mutation will cause loss of hydrophobic interactions in the core of the protein. |
| L90F | 2^nd^ transmembrane domain | 8 | extended strand | W<M |  |  | The wild-type residue was buried in the core of the protein. The mutant residue is bigger and probably will not fit. |
| G94A | Topological domain, cytoplasmic, intracellular loop | 9 | α helix | W<M |  | ↑ | The residue is located on the surface of the protein, mutation of this residue can disturb interactions with other molecules or other parts of the protein. The torsion angles for this residue are unusual. Only glycine is flexible enough to make these torsion angles, mutation into another residue will force the local backbone into an incorrect conformation and will disturb the local structure. |
| H98Q | Topological domain, cytoplasmic, intracellular loop | 9 | α helix | W>M |  |  | This will cause a possible loss of external interactions. |
| R101P | Topological domain, cytoplasmic, intracellular loop | 8 | α helix | W>M | Pos→Neu | ↑ | The charge of the wild-type residue is lost by this mutation. This can cause loss of interactions with other molecules. |
| G143R | Topological domain, cytoplasmic, intracellular loop | 8 | β turn | W<M | Neu→Pos | ↓ | The mutation introduces a charge at this position, this can cause repulsion between the mutant residue and neighboring residues. The residue is located on the surface of the protein, mutation of this residue can disturb interactions with other molecules or other parts of the protein. The torsion angles for this residue are unusual. Only glycine is flexible enough to make these torsion angles, mutation into another residue will force the local backbone into an incorrect conformation and will disturb the local structure. |
| G143E | Topological domain, cytoplasmic, intracellular loop | 8 | β turn | W<M | Neu→Neg | ↓ | The mutation introduces a charge at this position, this can cause repulsion between the mutant residue and neighboring residues. The residue is located on the surface of the protein, mutation of this residue can disturb interactions with other molecules or other parts of the protein. The torsion angles for this residue are unusual. Only glycine is flexible enough to make these torsion angles, mutation into another residue will force the local backbone into an incorrect conformation and will disturb the local structure. |
| L146R | Topological domain, cytoplasmic, intracellular loop | 9 | α helix | W<M | Neu→Pos | ↓ | The mutation introduces a charge at this position, this can cause repulsion between the mutant residue and neighboring residues. The residue is located on the surface of the protein, mutation of this residue can disturb interactions with other molecules or other parts of the protein. The mutation might cause loss of hydrophobic interactions with other molecules on the surface of the protein. |
| R147Q | Topological domain, cytoplasmic, intracellular loop | 6 | α helix | W>M | Pos→Neu |  | The charge of the wild-type residue is lost by this mutation. This can cause loss of interactions with other molecules. This will cause a possible loss of external interactions. |
| R147W | Topological domain, cytoplasmic, intracellular loop | 6 | α helix | W<M | Pos→Neu | ↑ | The charge of the wild-type residue is lost by this mutation. This can cause loss of interactions with other molecules. The residue is located on the surface of the protein, mutation of this residue can disturb interactions with other molecules or other parts of the protein. |
| T148I | Topological domain, cytoplasmic, intracellular loop | 9 | α helix | W<M |  | ↑ | The residue is located on the surface of the protein, mutation of this residue can disturb interactions with other molecules or other parts of the protein. |
| N152I | Topological domain, cytoplasmic, intracellular loop | 9 | α helix | W>M |  | ↑ | The mutation will cause an empty space in the core of the protein. The mutation will cause loss of hydrogen bonds in the core of the protein and as a result disturb correct folding. |
| F155V | 3^rd^ transmembrane domain | 7 | α helix | W>M |  |  | This will cause a possible loss of external interactions. |
| K156Q | 3^rd^ transmembrane domain | 9 | α helix | W>M | Pos→Neu |  | The charge of the buried wild-type residue is lost by this mutation. The mutation will cause an empty space in the core of the protein. |
| T157K | 3^rd^ transmembrane domain | 8 | α helix | W<M | Neu→Pos | ↓ | The mutant residue introduces a charge in a buried residue which can lead to protein folding problems. The wild-type residue was buried in the core of the protein. The mutant residue is bigger and probably will not fit. The mutation will cause loss of hydrophobic interactions in the core of the protein. |
| G162C | 3^rd^ transmembrane domain | 6 | β turn | W<M |  | ↑ | The residue is located on the surface of the protein, mutation of this residue can disturb interactions with other molecules or other parts of the protein. The torsion angles for this residue are unusual. Only glycine is flexible enough to make these torsion angles, mutation into another residue will force the local backbone into an incorrect conformation and will disturb the local structure. |
| G162S | 3^rd^ transmembrane domain | 6 | β turn | W<M |  |  | The residue is located on the surface of the protein, mutation of this residue can disturb interactions with other molecules or other parts of the protein. The torsion angles for this residue are unusual. Only glycine is flexible enough to make these torsion angles, mutation into another residue will force the local backbone into an incorrect conformation and will disturb the local structure. |
| Q167R | 3^rd^ transmembrane domain | 9 | random coil | W<M | Neu→Pos |  | The mutant residue introduces a charge in a buried residue which can lead to protein folding problems. The wild-type residue was buried in the core of the protein. The mutant residue is bigger and probably will not fit. |
| G172D | 3^rd^ transmembrane domain | 9 | β turn | W<M | Neu→Neg | ↓ | The mutation introduces a charge at this position, this can cause repulsion between the mutant residue and neighboring residues. The residue is located on the surface of the protein, mutation of this residue can disturb interactions with other molecules or other parts of the protein. The torsion angles for this residue are unusual. Only glycine is flexible enough to make these torsion angles, mutation into another residue will force the local backbone into an incorrect conformation and will disturb the local structure. |
| G172S | 3^rd^ transmembrane domain | 9 | β turn | W<M |  |  | The residue is located on the surface of the protein, mutation of this residue can disturb interactions with other molecules or other parts of the protein. The torsion angles for this residue are unusual. Only glycine is flexible enough to make these torsion angles, mutation into another residue will force the local backbone into an incorrect conformation and will disturb the local structure. |
| F173L | 3^rd^ transmembrane domain | 8 | random coil | W>M |  |  | This will cause a possible loss of external interactions. |
| P177R | 2^nd^ extracellular loop | 8 | random coil | W<M | Neu→Pos | ↓ | The mutation introduces a charge at this position, this can cause repulsion between the mutant residue and neighboring residues. The residue is located on the surface of the protein, mutation of this residue can disturb interactions with other molecules or other parts of the protein. The mutation might cause loss of hydrophobic interactions with other molecules on the surface of the protein. |
| P177L | 2^nd^ extracellular loop | 8 | random coil | W<M |  |  | The residue is located on the surface of the protein, mutation of this residue can disturb interactions with other molecules or other parts of the protein. |
| R180G | 2^nd^ extracellular loop | 5 | random coil | W>M | Pos→Neu | ↑ | This will cause a possible loss of external interactions. |
| R183G | 2^nd^ extracellular loop | 7 | random coil | W>M | Pos→Neu | ↑ | This can cause loss of interactions with other molecules. |
| P187L | 2^nd^ extracellular loop | 9 | random coil | W<M |  |  | The residue is located on the surface of the protein, mutation of this residue can disturb interactions with other molecules or other parts of the protein. |
| P187S | 2^nd^ extracellular loop | 9 | random coil | W>M |  | ↓ | This will cause a possible loss of external interactions. The mutation might cause loss of hydrophobic interactions with other molecules on the surface of the protein. |
| N188I | 2^nd^ extracellular loop | 8 | random coil | W>M |  | ↑ | This will cause a possible loss of external interactions. |
| N188S | 2^nd^ extracellular loop | 8 | random coil | W>M |  | ↑ | This will cause a possible loss of external interactions. |
| N188T | 2^nd^ extracellular loop | 8 | random coil | W>M |  | ↑ | This will cause a possible loss of external interactions. |
| V190G | 2^nd^ extracellular loop | 9 | extended strand | W>M |  | ↓ | The mutation will cause an empty space in the core of the protein. The mutation will cause loss of hydrophobic interactions in the core of the protein. |
| F193L | 2^nd^ extracellular loop | 8 | extended strand | W>M |  |  | The mutation will cause an empty space in the core of the protein. |
| F193S | 2^nd^ extracellular loop | 8 | extended strand | W>M |  | ↓ | The mutation will cause an empty space in the core of the protein. The mutation will cause loss of hydrophobic interactions in the core of the protein. |
| S195F | 2^nd^ extracellular loop | 9 | random coil | W<M |  | ↑ | The wild-type residue was buried in the core of the protein. The mutant residue is bigger and probably will not fit. The mutation will cause loss of hydrogen bonds in the core of the protein and as a result disturb correct folding. |
| P197S | 2^nd^ extracellular loop | 9 | random coil | W>M |  | ↓ | The mutation will cause an empty space in the core of the protein. The mutation will cause loss of hydrophobic interactions in the core of the protein. |
| T198M | 2^nd^ extracellular loop | 9 | random coil | W<M |  | ↑ | The residue is located on the surface of the protein, mutation of this residue can disturb interactions with other molecules or other parts of the protein. |
| E199A | 2^nd^ extracellular loop | 9 | random coil | W>M | Neg→Neu | ↑ | The mutation will cause an empty space in the core of the protein. The mutation will cause loss of hydrophobic interactions in the core of the protein. |
| F206I | 4^th^ transmembrane domain | 9 | extended strand | W>M |  |  | This will cause a possible loss of external interactions. |
| M207L | 4^rd^ transmembrane domain | 9 | extended strand | W>M |  |  | The mutation will cause an empty space in the core of the protein. |
| A211V | 4^th^ transmembrane domain | 9 | α helix | W<M |  |  | The wild-type residue was buried in the core of the protein. The mutant residue is bigger and probably will not fit. |
| L220Q | 4^th^ transmembrane domain | 6 | α helix | W<M |  | ↓ | The residue is located on the surface of the protein, mutation of this residue can disturb interactions with other molecules or other parts of the protein. The mutation might cause loss of hydrophobic interactions with other molecules on the surface of the protein. |
| W227R | Intramembrane, COOH_2_-termini | 4 | α helix | W>M | Neu→Pos | ↓ | The mutation introduces a charge, this can cause repulsion of ligands or other residues with the same charge. Hydrophobic interactions, either in the core of the protein or on the surface, will be lost. |
| N327K | Intramembrane, COOH_2_-termini | 7 | α helix | W<M | Neu→Pos |  | The mutation introduces a charge, this can cause repulsion of ligands or other residues with the same charge. |
